# Progress toward a Universal H5N1 Vaccine: A Recombinant Modified Vaccinia Virus Ankara-Expressing Trivalent Hemagglutinin Vaccine

**DOI:** 10.1371/journal.pone.0107316

**Published:** 2014-09-17

**Authors:** Mookkan Prabakaran, Sonja Leyrer, Fang He, Sebastian Auer, Subaschandrabose R. Kumar, Kathrin Kindsmueller, Nutan Mytle, Joerg Schneider, Stephen Lockhart, Jimmy Kwang

**Affiliations:** 1 Animal Health Biotechnology, Temasek Life Sciences Laboratory, National University of Singapore, Singapore, Singapore; 2 Emergent BioSolutions Inc., Rockville, Maryland, United States of America; Lindsley F. Kimball Research Institute, United States of America

## Abstract

**Background:**

The rapid evolution of new sublineages of H5N1 influenza poses the greatest challenge in control of H5N1 infection by currently existing vaccines. To overcome this, an MVAtor vector expressing three H5HA antigens A/Vietnam/1203/04, A/Indonesia/669/06 and A/Anhui/01/05 (MVAtor-tri-HA vector) was developed to elicit broad cross-protection against diverse clades by covering amino acid variations in the major neutralizing epitopes of HA among H5N1 subtypes.

**Methods:**

BALB/c mice and guinea pigs were immunized i.m. with 8×10^7^ TCID_50_/animal of MVAtor-tri-HA vector. The immunogenicity and cross-protective immunity of the MVAtor-tri-HA vector was evaluated against diverse clades of H5N1 strains.

**Results:**

The results showed that mice immunized with MVAtor-tri-HA vector induced robust cross-neutralizing immunity to diverse H5N1 clades. In addition, the MVAtor-tri-HA vector completely protected against 10 MLD_50_ of a divergent clade of H5N1 infection (clade 7). Importantly, the serological surveillance of post-vaccinated guinea pig sera demonstrated that MVAtor-tri-HA vector was able to elicit strong cross-clade neutralizing immunity against twenty different H5N1 strains from six clades that emerged between 1997 and 2012.

**Conclusions:**

The present findings revealed that incorporation of carefully selected HA genes from divergent H5N1 strains within a single vector could be an effective approach in developing a vaccine with broad coverage to prevent infection during a pandemic situation.

## Introduction

The continuing evolution of highly pathogenic H5N1 avian influenza in Asia and the recent emergence of H7N9 avian influenza in humans in Eastern China are increasing the threat of the next influenza pandemic. As of January 2014, the World Health Organization confirmed 650 human cases of H5N1 infection with 386 deaths [1]. Control of infection with current H5N1 vaccines dose not appear to be effective against heterologous strains or variant clades of H5N1 due to variation in the globular head of hemagglutinin (HA). As the H5N1 viruses are composed of 10 different clades and multiple subclades, the development of a universal H5N1 vaccine for pandemic preparedness has been severely hampered. Our approach to overcome the antigenic diversity of H5N1 influenza virus clades focuses on the design of multivalent vaccines based on the distribution of major neutralizing epitopes in the globular head of HA, the principal determinants of protective immunity to influenza virus. Previously, we identified three such vaccine strains, A/Vietnam/1203/04 (clade 1), A/Indonesia/CDC669/06 (clade 2.1.3.2) and A/Anhui/01/05 (clade 2.3.4) to cover most of the variations in the neutralizing epitopes of H5N1 lineages [2]. The HAs of those selected vaccine strains were individually expressed on the baculovirus surface (BacHA) and the cross-protective efficacy of a trivalent BacHA combination confirmed in a mouse model [2]. In this study we have investigated an approach to enhance the neutralizing efficacy against a wide variety of H5N1 strains, including circulating H5N1 strains, while combining HAs in a single vector to reduce the vaccine dose and avoid the complexity of vaccine production, testing and formulation.

Progressively developing recombinant vector technologies can efficiently deliver multiple genes in a single vector, which allows the cost-effective production of large quantities in a single manufactured product. Vectored vaccines based on adenovirus and poxviruses are among the several human viruses that have been extensively exploited for the development of multivalent vector-based vaccines [3]–[5]. Among the poxviruses, the replication-deficient modified vaccinia virus Ankara (MVA) vector is an attractive vaccine production platform based on its well-documented safety profile and potent immunogenicity capabilities as demonstrated in several clinical trials and vaccination of more than 120,000 humans [6]. Hence, we used MVAtor (modified vaccinia virus Ankara vector) a derivative of the pre-vaccine used for the smallpox eradication campaign in Germany in the early 1980s as a vaccine vector to express selected HA genes efficiently in a single recombinant construct.

In order to obtain proof of concept, selected HA genes from three H5N1 strains were inserted into recombinant MVAtor vector in a single insertion site (MVAtor-tri-HA) and the cross-protective efficacy of the vaccine candidate was evaluated in a mouse model. Additionally, serological surveillance was conducted to evaluate the neutralizing efficacy of post-vaccinated guinea pig sera against various clades of H5N1 strains that circulated worldwide during 1997–2012.

## Materials and Methods

### Ethics

All animal experiments were reviewed and approved by the Institutional Animal Care and Use Committee (IACUC) of the Temasek Life Sciences Laboratory, Singapore. (IACUC approval numbers TLL-EB-10-004, TLL-EB-12-001, TLL-11-012). All challenge experiments were conducted in a animal BSL3 containment facility in compliance with CDC/NIH and WHO recommendations.

### Viruses and cells

H5N1 virus A/Chicken/Cambodia/008LC1/2011 (clade 1.1) was obtained from the National Influenza Centre, Institute Pasteur in Cambodia. The hemagglutinin (HA) and neuraminidase (NA) genes of H5N1 viruses from clades 0, 1, 2, 4, 7 and 9 (indicated by RG-H5N1 in Table 1) were synthesized (GenScript, USA) based on the sequence from the NCBI Influenza database. A reassortant virus containing the HA and NA from each H5N1 virus and the internal genes from A/Puerto Rico/8/1934 was generated [7], [8]. Virus titer was determined by hemagglutination assay as described previously [9]. Chicken embryo fibroblast cells (CEF) were obtained from 11 day-old fertilized specific-pathogen free chicken eggs (Lohmann Tierzucht GmbH, Germany). MVAtor vector was derived from MVA470 StMUG from the Bavarian State Office for Health and Food Safety, Germany. All experiments with highly pathogenic viruses were conducted in a biosafety level 3 (BSL3) containment facility in compliance with CDC/NIH recommendations [10].

**Table 1:** Serological surveillance of anti-MVAtor-tri-HA sera against H5N1 strains by HAI and VMN titers.

H5N1 Viruses	Clade	Host	Year	HAI titer	VMN titer
RG-VN/1203/2004* #	1	Human	2004	512	1280
Indo/CDC669/2006*	2.1.3.2	Human	2006	512	1280
RG-Anhui/01/2005* #	2.3.4	Human	2005	256	640
RG-A/Hongkong/156/1997#	0	Human	1997	1024	1280
RG-A/HongKong/213/2003	1	Human	2003	1024	1280
RG-A/duck/Thailand/CV-328/2007	1	Avian	2007	512	1280
A/Chicken/Cambodia/008LC1/2011	1.1	Avian	2011	512	1280
A/Indonesia/CDC1031/2007	2.1.3.2	Human	2007	512	1280
RG-A/Turkey/Turkey1/2005#	2.2.1	Avian	2005	256	320
RG-A/Nigeria/6e/2007	2.2	Human	2007	512	640
RG-A/muscovyduckRostovonDon/51/2007	2.2	Human	2007	512	640
A/Egypt/3300-NAMRU3/2008#	2.2	Human	2008	512	1280
RG- A/chicken/Egypt/10249SF/2010	2.2.1	Avian	2010	512	1280
RG- A/chicken/Egypt/12186F/2012	2.2.1.1	Avian	2012	512	640
RG-A/MuscovyduckVietnam/33/2007	2.3.2	Avian	2007	512	640
RG-A/Whooper Swan/Akita/1/2008	2.3.2.1	Avian	2008	256	320
RG-A/bar-headed goose/Qinghai/1/2010	2.3.2.1	Avian	2010	256	640
RG-A/Hubei/1/2010#	2.3.2.1	Human	2010	512	640
RG-A/chicken/Nongkhai/NIAH400802/2007	2.3.4	Human	2007	512	640
RG-A/goose/Guiyang/337/2006#	4	Avian	2006	512	640
RG-A/chicken/Shanxi/2/2006	7	Avian	2006	512	640
RG- A/chicken/Vietnam/NCVD-03/2008#	7.1	Avian	2008	256	320
RG-A/chicken/Henan/12/2004	9	Avian	2004	256	640

*Trivalent vaccine strains (A/Vietnam/1203/04, A/Indonesia/CDC669/06 & A/Anhui/01/05).

#WHO recommended H5N1 vaccine strains.

### Selection of vaccine strains

The HA genes of three different H5N1 strains (Vietnam/1203/04, A/Indonesia/CDC669/06 and A/Anhui/01/05) were previously selected in a three-step approach: mapping of neutralizing epitopes of hemagglutinin by using neutralizing monoclonal antibodies (mAbs), an analysis of the distribution of identified neutralizing epitopes among all H5N1 lineages, and the selection of vaccine strains to cover the variations within the neutralizing epitopes of H5N1 subtypes [2]. The selected hemagglutinin genes of three H5N1 strains were inserted into MVAtor to generate MVAtor-tri-HA vector as described bellow.

### Construction and generation of MVAtor-tri-HA vector

**Figure 1:**
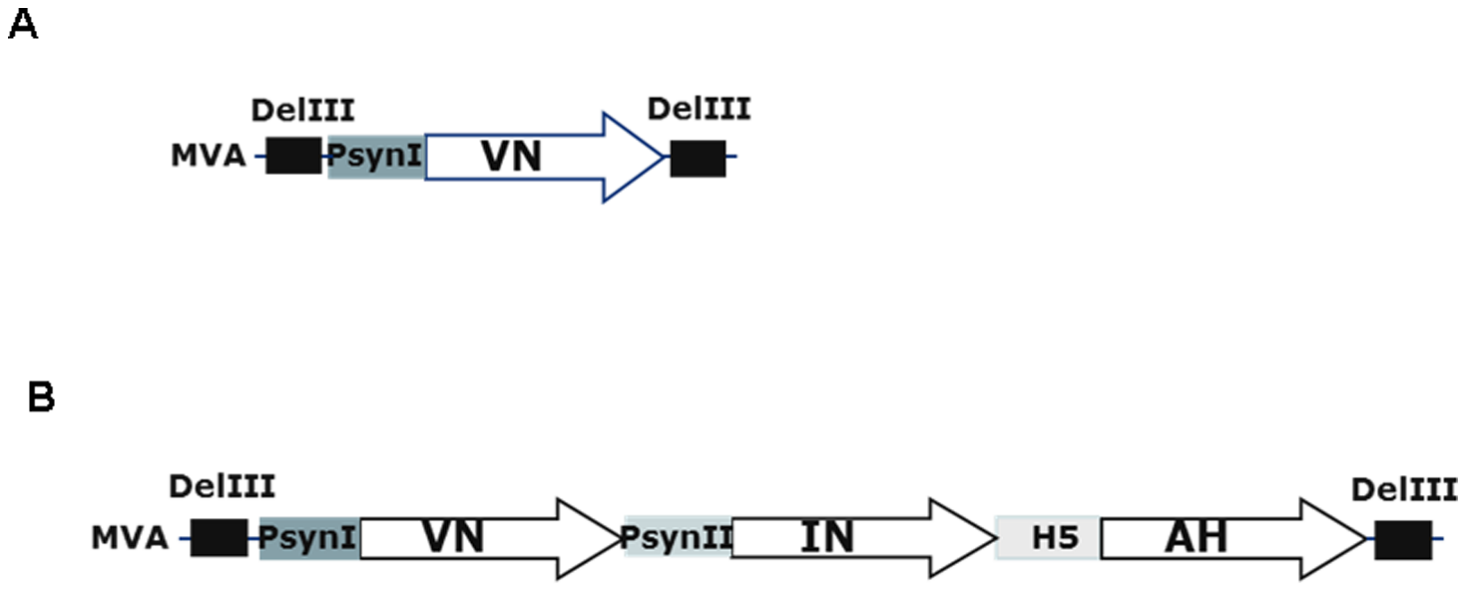
Schematic representation of recombinant MVAtor genomes. HA gene of A/Vietnam/1203/04 (MVAtor-mono-HA) (A) or HA genes of A/Vietnam/1203/04 (VN), A/Indonesia/CDC669/06 (IN), A/Anhui/01/05 (AH) (MVAtor-tri-HA) (B). Abbreviations: DelIII  =  deletion III; PsynI  =  synthetic promoter I; PsynII  =  synthetic promoter II; H5  =  H5 promoter. Arrows indicate the direction of transcription.

For the construction of the MVAtor-tri-HA vector the sequences were optimized for minimal homology and the three HAs from A/Vietnam/1203/04, A/Indonesia/CDC669/06 and A/Anhui/01/05 were fused to the PsynI, PsynII and H5 promoters, respectively within one DNA fragment (synthesized by Geneart) (Fig. 1). The complete transgenic cassette incorporating the three HAs was cloned into the KasI/HindIII restriction sites of the plasmid vector vEM11 by deletion of the lacZ gene for blue/white selection in competent E. coli bacteria, generating the shuttle vector vEM106. For the construction of the MVAtor-mono-HA vector, the HA gene of A/Vietnam/1203/04 was amplified and inserted along with PsynI promoter into the KasI/XhoI restriction sites of plasmid vector vEM11 generating the shuttle vector vEM99.

The shuttle vectors vEM99 and vEM106 were generated by direct insertion of HA genes in deletion III within the MVA genome. CEF cells were infected with MVAtor vector with a multiplicity of infection of 0.05 for one hour at room temperature. Then the cells were transfected with shuttle vector vEM99 or vEM106 using FuGene HD (Promega, USA). The homologous recombination resulted in MVAtor-mono-HA or MVAtor-tri-HA vector. After plaque selection on CEF cells the recombinant MVAtor constructs were analyzed by PCR to verify HA gene insertion and lack of residual empty vector. To generate virus stocks/POC material the viruses were amplified in CEF cells and purified by ultracentrifugation through a sucrose cushion and reconstituted in 25 mM Tris buffer (pH 9.0). Original stock of the control virus MVA-LacZ was kindly provided by Prof. Gerd Sutter (Ludwig-Maximilian University of Munich, Germany).

### Immunofluorescence assay to detect individual HA expression

HeLa cells infected with MVAtor-mono-HA or MVAtor-tri-HA vector were analysed by immunofluorescence using monoclonal antibodies (mAbs) specific for each HA, anti-AH for Anhui HA (Sino Biological, China), 3H11 [11] and 11G12 [12] for Indonesia HA, Vietnam HA, respectively. Briefly, HeLa cells in a μ-slide VI 0.4 (Ibidi) were infected with recombinant MVA and incubated for 24 hours at 37°C with 5% CO_2_. Cells were incubated for another 20–24 hours. After fixation, cells were permeabilized with 0.1% Triton X-100 and stained using the HA specific mAbs or vaccinia virus specific antibodies. The cells were then incubated with Alexa Fluor-conjugated anti-mouse antibody (Invitrogen, USA) and DAPI (Th. Geyer, Germany) and analysed with a fluorescence microscope.

### Mice immunization and challenge

Female BALB/c mice aged 6–7 weeks were purchased from Biological Resource centre, A Star Research institute, Singapore and housed at the Animal Holding Unit of the Temasek Life Sciences Laboratory, Singapore. Mice (40 mice per group) were immunized intramuscularly (i.m.) with 100 µL of 8×10^7^ TCID_50_ of MVAtor-tri-HA vector or MVA-LacZ control vector on day 0 and day 28. In separate studies, the reference control group (n = 40 mice) was immunized subcutaneously (s.c.) with 100 µL of 256 HA units of inactive whole H5N1 virus (A/Indonesia/CDC669/06), emulsified with Montanide ISA563 adjuvant. The H5N1 virus was inactivated with binary ethylenimine as described previously [13]. The monovalent control group (n = 10 mice) was immunized i.m. with 100 µL of 8×10^7^ TCID_50_ of monovalent MVAtor-HA (A/Vietnam/1203/04) vector. Blood samples were collected on days 27 and 47 for serum hemagglutination inhibition (HAI) assay and virus micro-neutralization (VMN) assay.

To assess the protective efficacy of the vaccines, twenty mice from the MVAtor-tri-HA or MVA-LacZ vector or the inactive H5N1 vaccinated group were anesthetized intraperitoneally with ketamine (100 mg/kg)/Xylazine (20 mg/kg) and intranasally challenged with 50 µL (25 µL per naris) of 10 MLD_50_ of A/Vietnam/1203/04 (clade 1) or A/chicken/Shanxi/2/06 (clade 7) H5N1 strain on day 49. Mice were observed daily to monitor body weight, clinical signs of disease (ruffled fur, lack of mobility, laboured breathing) and mortality. Mice were humanely euthanized with CO_2_ inhalation if their body weight dropped to 75% of baseline weights. For determination of lung viral titers after challenge, five mice from each vaccinated group were euthanized by CO_2_ inhalation on day 3 post-challenge. Whole lungs were homogenized in 1 mL Dulbecco's Minimal Essential Medium to enable 10-fold serially diluted suspensions of lung samples. The homogenized suspensions were titrated on monolayers of MDCK cells. The viral titers were calculated [14] and expressed as log_10_ TCID_50_/mL ± S.E. The limit of virus detection was 1.5 log_10_ TCID_50_/mL of lung tissue specimen.

### Serum HAI Assay

Receptor-destroying enzyme (Denka Seiken, Japan) treated sera were serially diluted two fold in V-bottom 96-well plates. Four hemagglutination units of each influenza viral antigen was added to each well of the 96-well plate and incubated with the serum for 30 min and 1% chicken red blood cells were added, and incubated for 40 min at room temperature [9].

### Micro-neutralization Assay

Serum VMN antibody titer was performed as described previously [15]. Serial two-fold dilutions of heat-inactivated (56°C for 30 min) immune sera were mixed separately with 100×50% tissue culture infective dose (TCID_50_) of H5N1 virus and incubated at room temperature for 1 h. The virus antibody mixture was then added to the monolayer of MDCK cells and incubated for 72 h at 37°C. The neutralizing antibody titer was expressed as the highest dilution of serum which showed no cytopathic effect.

### Generation of guinea pig anti-MVAtor-tri-HA sera for serological surveillance study

The objective of the serological surveillance study was to confirm the broadly neutralizing activity of the immune sera against distinct clades/subclades of H5N1 strains that emerged and circulated worldwide during 1997–2012. Female, Dunkin-Hartley Guinea Pigs (n = 10) were immunized i.m. three times on day 0, 28 and 49 with 100 µL of 8×10^7^ TCID_50_/animal of MVAtor-tri-HA vector. Additionally, two animals were immunized with MVA-LacZ as a negative control. After the final immunization, all the sera were individually tested for neutralizing antibody titer. The sera were pooled into large aliquots and stored at −20°C for further serological surveillance. Sera were tested for antibodies by HAI and VMN titers against clade 1, 2.1, 2.2, 2.3, 4, 7, 7.1 and 9, that emerged and circulated worldwide since 1997 (Table 1).

### Statistical analysis

The data were expressed as geometric or arithmetic mean as indicated ± standard deviation (SD) standard error (SE). The unpaired two tailed Student's *t*-test was performed to determine the level of significance in the difference between means of two groups. The level of significance was expressed as *P*<0.05.

## Results

### Trivalent MVA vaccine candidate construction and characterization

**Figure 2:**
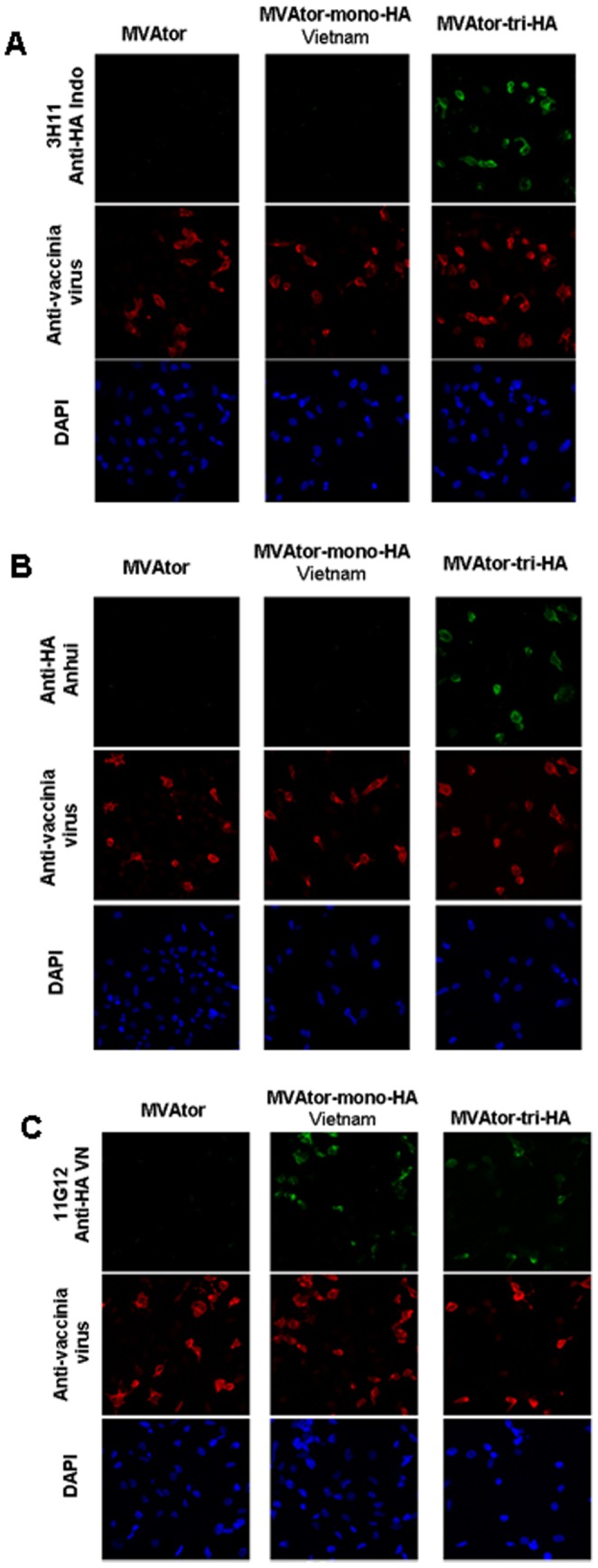
Immunofluorescence of recombinant MVAtor vector infected cells. HeLa cells were infected with MVAtor-mono-HA or MVAtor-tri-HA or MVAtor empty vector respectively and stained with 3H11 mAb specific against A/Indonesia/CDC669/06 (A), AH mAb specific against A/Anhui/01/05 H5N1 strain (B), 11G12 mAb specific against A/1203/04 H5N1 strain (C). Vaccinia virus specific antibodies (anti-Vaccinia Virus) and DAPI used as controls. Magnification 20 x.

Recombinant MVAtor vector expressing the Vietnam HA gene (MVAtor-mono-HA) and the three HA proteins (MVAtor-tri-HA) were generated by integration of the HA expression cassettes into the deletion III site of the MVA genome by homologous recombination (Fig. 1). The MVAtor vectors were propagated on CEF cells, purified and their titers adjusted to 8×10^7^ TCID_50_/mL. The purified stock was analyzed by PCR (data not shown) and titrated in CEF cells (Fig. 2). Also, the immunofluorescence assay against individual HA-specific antibodies demonstrated efficient expression of all three HAs by a single MVAtor-tri-HA vector (Fig 2A, B & C). In contrast, no fluorescent cells were seen for the MVA negative control (Fig. 2: MVAtor; all HA antibodies).

### Determination of HAI titer against MVAtor-tri-HA vector in mice

**Figure 3:**
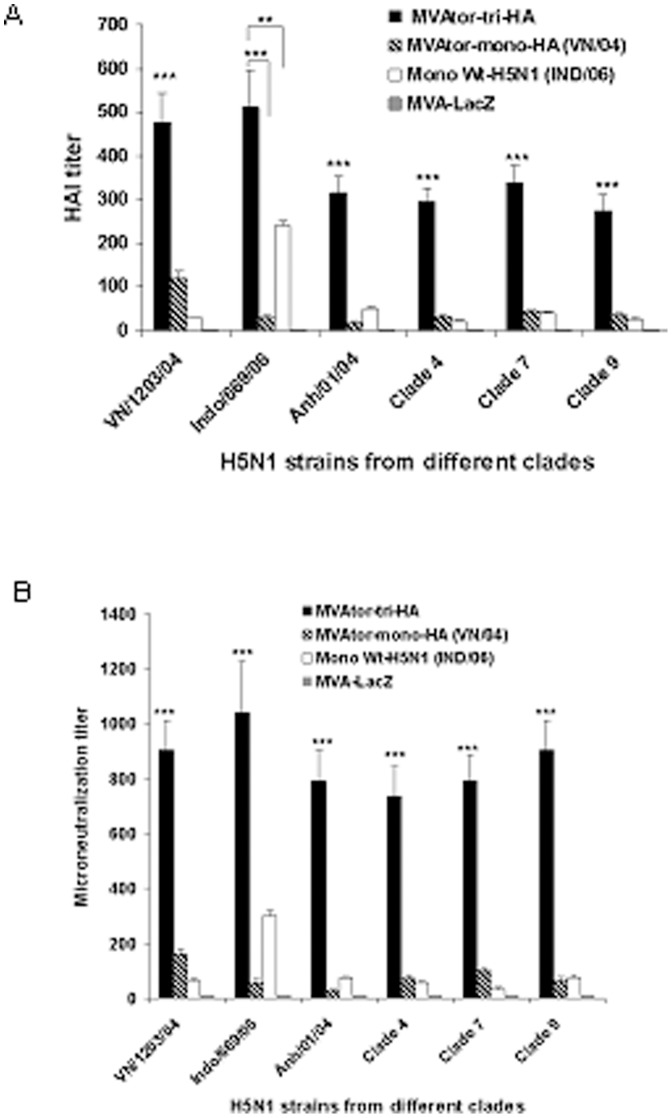
HAI and VMN titers against H5N1 strains. The specific immune responses of mice immunized i.m. on days 0 and 28 with MVAtor-tri-HA or MVAtor-mono-HA (A/Vietnam/1203/04) or MVA-LacZ vector. A reference group of mice was vaccinated s.c. with adjuvanted inactivate H5N1 viral vaccine (A/Indonesia/CDC669/06). Serum hemagglutination inhibition (HAI) assay (A). The serum HAI titer on day 47 against homologous H5N1 strains, clade 1 (RG-A/Vietnam/1203/04), clade 2.1.3.2 (A/Indonesia/CDC669/06), clade 2.3.4 (RG-A/Anhui/01/2005) and heterologous H5N1 strains from clade 4 (RG-A/goose/Guiyang/337/2006), clade 7 (RG-A/chicken/Shanxi/2/2006) and clade 9 (RG-A/chicken/Henan/12/2004). Each point represents the geometric mean titer (n = 10) ± SE. (**P<0.01; ***P<0.001). Serum virus microneutralization (VMN) assay on day 47 against homologous and heterologous H5N1 strains (B). Each point represents the geometric mean titer (n = 10) ± standard error (SE). (***P<0.001).

Hemagglutination inhibition titers measure the antibody response to inhibit HA function. As shown in Figure 3A, the sera from mice immunized with MVAtor-tri-HA vector efficiently induced HAI titer against homologous viruses (1∶480 against A/Vietnam/1203/04, 1∶510 against A/Indonesia/CDC669/06 and 1∶315 against A/Anhui/01/05) on day 47. In comparison, mice vaccinated with monovalent inactive H5N1 (A/Indonesia/CDC669/06) vaccine and MVAtor-mono-HA vector (A/Vietnam/1203/04) showed HAI titers against the homologous H5N1 strain of 1∶240 and 1∶120, respectively. Importantly, mice immunized with MVAtor-tri-HA vector showed significantly increased HAI titers against heterologous clades of H5N1 strains (1∶290 against clade 4, 1∶340 against clade 7 and 1∶270 against clade 9; P<0.001), compared to MVAtor-mono-HA vector or adjuvanted monovalent inactive H5N1 vaccine, which showed very low HAI titer (<1∶42) against heterologous clades of H5N1 strains (Fig. 3A).

### Induction of cross-neutralizing antibodies against MVAtor-tri-HA vector

VMN assay was performed to determine functional antibodies responsible for the protective immunity against influenza. The serum neutralizing antibody titer against 100 TCID_50_ of different clades of H5N1 strains on day 47 showed that vaccination with MVAtor-tri-HA vector led to a significant increase (P<0.001) in neutralizing antibody titers against homologous H5N1 strains (1∶905 against A/Vietnam/1203/04, 1∶1040 against A/Indonesia/CDC669/06 and 1∶790 against A/Anhui/01/05) compared to mice immunized with MVAtor-mono-HA vector (A/Vietnam/1203/04) or monovalent inactive H5N1 (A/Indonesia/CDC669/06) vaccine. Importantly, mice immunized with MVAtor-tri-HA vector showed cross-neutralizing antibodies against different clades of H5N1 strains (1∶735 against clade 4, 1∶780 against clade 7 and 1∶905 against clade 9) (Fig. 3B). In contrast, mice immunized s.c. with monovalent inactive whole H5N1 vaccine developed a neutralizing antibody response against homologous H5N1 strain (1∶280), but inactive H5N1 vaccine did not induce efficient neutralizing antibody response against heterologous clades of H5N1 strains (Fig. 3B).

### Protection against 10 MLD_50_ of distinct H5N1 viral challenge

**Figure 4:**
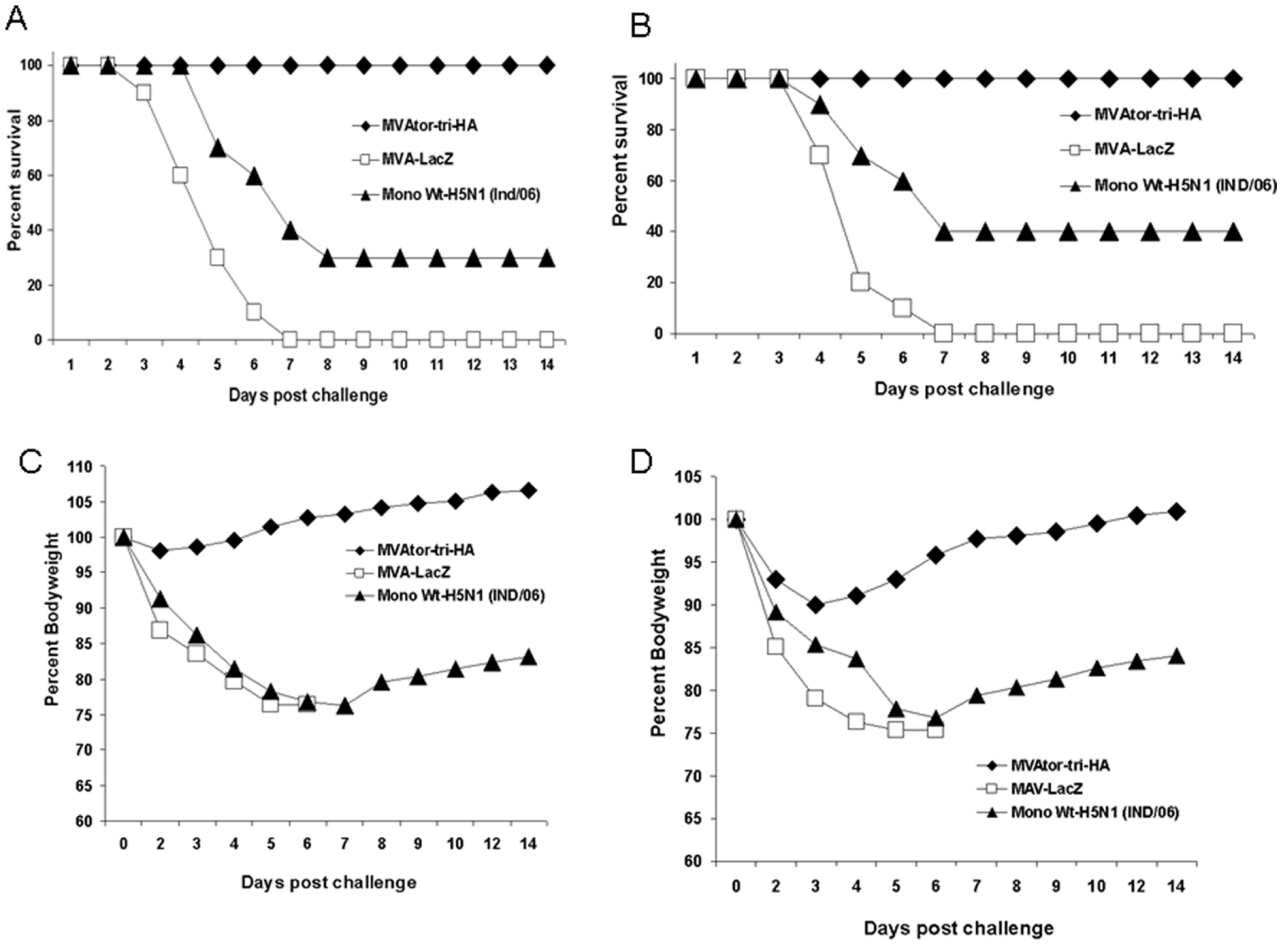
Protection of mice from lethal H5N1 virus challenge. Each group of mice was immunized i.m. two times on days 0 and 28 with MVAtor-tri-HA or MVAtor-mono-HA (A/Vietnam/1203/04) or MVA-LacZ vector. The reference control group was immunized s.c. with adjuvanted inactive H5N1 viral vaccine (A/Indonesia/CDC669/06). Three weeks after the second vaccination, mice were intranasally infected with 10 MLD_50_ of a clade 1.0 (RG-A/Vietnam/1203/04) or clade 7.0 (RG-A/chicken/Shanxi/2/2006) H5N1 strain. Mice were monitored for survival throughout a 14-day observation period after clade 1.0 (A) or clade 7.0 (B) H5N1 viral challenge. The results are expressed in percent survival. Mice were monitored for weight loss throughout a 14-day observation period after clade 1.0 (C) or clade 7.0 (D) challenge. The results are expressed in terms of percent body weight compared to the start of the viral challenge.

The cross-protective efficacy of MVAtor-tri-HA vector in mice was tested in an in vivo challenge experiment. Three weeks after the second immunization, groups of mice were challenged with 10 MLD_50_ of highly pathogenic homologous, clade 1 (RG-A/Vietnam/1203/04) or heterologous, clade 7 (RG-A/chicken/Shanxi/2/06) H5N1 virus. Mice immunized with MVAtor-tri-HA vector obtained complete protection from weight loss and death against clade 1 challenge while adjuvanted inactive whole H5N1 virus (A/Indonesia/CDC669/06) vaccinated mice rapidly lost body weight and showed only 30% protection (Fig 4A and C). In addition, MVAtor-tri-HA vector immunized mice displayed no signs of disease after clade 1 challenge, while MVA-LacZ or inactive whole H5N1 immunized animals developed clinical symptoms such as ruffled fur, laboured breathing and lack of mobility. Importantly, mice immunized with MVAtor-tri-HA vector showed 100% survival after infection with 10 MLD_50_ of a distinct clade 7 of H5N1 virus, and showed only mild decrease in body weight (up to 9%) on day 3 and regained their body weight rapidly (Fig. 4B and D). In contrast, mice which received the adjuvanted inactive whole H5N1 vaccine showed rapid decline in body weight with only 40% protection and severe clinical symptoms. Mice vaccinated with MVA-LacZ (negative control) died from complications associated with influenza infection or were euthanized when they lost ≥25% of their initial body weight.

**Figure 5:**
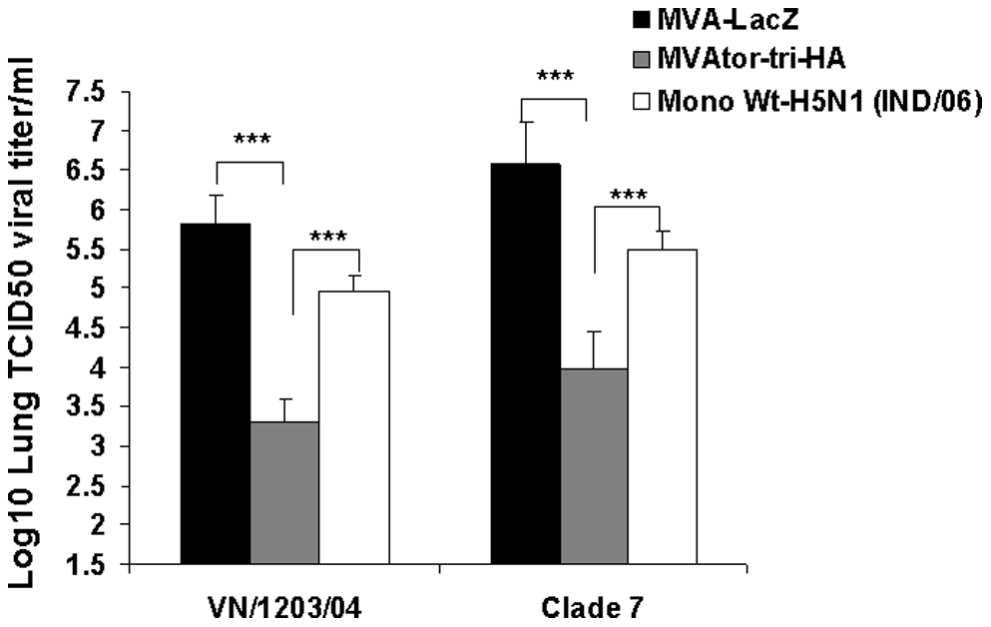
Virus titers in mouse lungs of experimentally infected with clade 1 or clade 7 H5N1 strain. Each group of mice was immunized i.m. two times on days 0 and 28 with MVAtor-tri-HA or MVAtor-mono-HA (A/Vietnam/1203/04) or MVA-LacZ vector. Three weeks after the second vaccination, mice were intranasally infected with 10 MLD_50_ of a clade 1.0 (RG-A/Vietnam/1203/04) or clade 7.0 (RG-A/chicken/Shanxi/2/2006) H5N1 strain. The viral loads were measured in the lungs of the infected animals on day 3 post challenge. The results are expressed in terms of mean value of log TCID_50_/mL ± standard deviation (SD). The lower limit of detection was 1.5 log_10_ TCID_50_/mL. (**P<0.01; ***P<0.001).

Viral titers in lungs were analyzed to determine the replication of challenge virus on day 3 post challenge. As shown in Figure 5, mice immunized with MVAtor-tri-HA vector had significantly lower lung viral titers upon homologous (P<0.001) and heterologous (P<0.001) challenge when compared to mice immunized with inactive whole H5N1 virus or MVA-LacZ (Fig. 5).

### Serological surveillance of post-vaccinated guinea pig sera against H5N1 subtype

A serological surveillance study was performed to confirm the ability of MVAtor-tri-HA immune guinea pig sera to neutralize distinct clades/subclades of H5N1 strains that have emerged between 1997 and 2012. HAI titer of sera from guinea pigs vaccinated with MVAtor-tri-HA vector had efficiently induced HAI titer against clade 0, 1, 2.1, 2.2, 2.3, 4, 7, 7.1 and 9 (Table 1). Also, serum cross-neutralizing antibody titer showed that guinea pig anti-MVAtor-tri-HA sera efficiently induced cross-VMN titers against all the twenty distinct H5N1 strains (Table 1). Moreover, the results of cross-neutralization assay also revealed that MVAtor-tri-HA vector immunization induced VMN titers against H5N1 strains from clade 1.1, 2.2.1.1 and 2.3.2.1 (circulating strains from 2010–2012). VMN titers of 1∶1280 against both clade 1.1 (A/Chicken/Cambodia/008LC1/2011) and clade 2.2.1 (A/chicken/Egypt/10249SF/2010), and 1∶640 VMN titers against clade 2.2.1.1 (A/Egypt/12186F/2012), clade 2.3.2.1 (A/Hubei/1/2010) and clade 2.3.2.1 (A/bar-headed goose/Qinghai/1/2010) H5N1 strains were obtained (Table 1).

## Discussion

The rapid evolution of new sublineages of H5N1 influenza in Asia and Egypt poses the greatest challenge in control of influenza H5N1 infection by currently existing vaccines. Hence, a broadly protective vaccine could induce some degree of cross-protection against a future pandemic H5N1 strain. Several strategies have been evaluated in an effort to increase a broad cross-protective efficacy of the H5N1 vaccine [16]–[18]. Ducatez et al. [17] reported that ancestral HA sequences reconstructed based on a phylogenitic topology elicited cross-protective immunity against heterologous H5N1 clades. Previously, Chen et al. [16] reported that consensus-based H5 HA DNA vaccine protects mice against divergent H5N1 clades. However, ancestral and consensus-based antigen designs are intrinsically influenced by the input sequences which may not accurately reflect the genetic diversity of H5N1 influenza virus. A recent report has demonstrated that priming twice with H5 DNA vaccine followed by a booster with VLP vaccine were found to enhance the neutralizing antibodies against different clades of H5N1 strains emerged before 2007 [18]. However, DNA vaccine prime-boost immunizations would require several months for completion of multiple dose regimens and very complex strategies. Most of these above approaches have been shown to elicit more cross-reactive antibodies against limited clades/subclades or H5N1 clades/subclades emerged before 2007.

Understanding the distribution of the major neutralizing epitopes in the globular head of HA among the H5N1 subtype could help in the selection of ideal vaccine strains. Previously, the neutralizing conformational epitopes of HA1 were mapped by the characterization of escape mutants with neutralizing mAbs [2], [19]. The conformational neutralizing epitopes located at amino acids 138–141 and 162 in the antigenic site A, amino acids 151–156 and 189 in the antigenic site B (H5 numbering, excluding signal peptide) are located within the receptor binding site (Table 2) and are important in vaccine strain selection. Variations in these neutralizing epitopes may render the current H5N1 vaccines ineffective for the prevention of heterologous types of H5N1 strains. Previously, we selected three vaccine strains A/Vietnam/1203/04 (clade 1), A/Indonesia/CDC669/06 (clade 2.1.3) and A/Anhui/1/05 (clade 2.3.4) to cover major variations in the neutralizing epitopes of H5N1 lineages [2].

**Table 2:** Variations and conservation of all identified neutralizing epitopes of HA.

H5N1 viruses	Clade	138^b^	139^b^	140^b^	141^a^	151^a^	152^a^	154^a^	155^b^	156^a^	162^a^	183^a^	189^b^	218^b^	223^b^
**RG-VN/1203/2004** *	1	Q	G	K	S	I	K	D	S	T	R	D	K	K	S
**Indo/CDC669/2006** *	2.1.3.2	L	G	S	P	I	K	N	S	T	K	D	R	K	S
**RG-Anhui/01/2005** *	2.3.4	Q	G	T	P	I	K	N	N	T	R	D	K	K	S
**RG-A/Hongkong/156/1997**	0	L	G	R	S	I	K	N	S	A	R	D	K	K	S
**RG-A/HongKong/213/2003**	1	Q	G	K	S	I	K	N	N	A	R	D	R	K	N
**RG-A/duck/Thailand/CV-328/2007**	1	Q	G	K	S	I	K	N	S	T	R	D	K	K	S
**A/Chicken/Cambodia/008LC1/2011**	1.1	Q	G	K	S	I	K	N	S	T	R	D	K	K	S
**A/Indonesia/CDC1031/2007**	2.1.3.2	L	G	S	P	I	K	N	S	T	K	N	R	K	S
**RG-A/Turkey/Turkey1/2005**	2.2.1	Q	G	R	S	I	K	D	N	A	R	D	R	K	S
**RG-A/Nigeria/6e/2007**	2.2	Q	G	R	S	I	K	D	N	A	R	D	R	K	S
**RG-A/mduck/RostovonDon/51/2007**	2.2	Q	G	R	S	I	K	N	D	A	R	D	R	K	S
**RG-**	2.2	Q	G	G	P	I	K	N	N	T	K	D	R	K	S
**RG- A/chicken/Egypt/10249SF/2010**	2.2.1	Q	G	G	S	T	K	N	D	A	K	D	R	K	S
**RG- A/chicken/Egypt/12186F-9/2012**	2.2.1.1	Q	G	R	S	T	K	N	D	A	K	D	R	K	S
**RG-A/Muscovyduck Vietnam/33/2007**	2.3.2	Q	G	K	S	I	K	N	S	T	R	D	K	K	S
**RG-A/Whooper Swan/Akita/1/2008**	2.3.2.1	Q	G	N	S	I	K	N	N	A	K	D	R	K	S
**RG-A/bar-headed goose/Qinghai/1/2010**	2.3.2.1	Q	G	N	S	I	K	D	N	A	K	D	R	K	S
**RG-A/Hubei/1/2010**	2.3.2.1	Q	G	K	S	I	K	K	N	A	K	D	R	K	S
**RG-A/chicken/Nongkhai/NIAH400802/2007**	2.3.4	Q	G	T	P	I	K	N	N	T	R	D	K	K	S
**RG-A/goose/Guiyang/337/2006**	4	L	G	E	S	I	K	N	S	S	R	D	K	K	S
**RG-A/chicken/Shanxi/2/2006**	7	L	G	K	P	I	K	N	N	T	V	D	K	K	S
**RG- A/chicken/Vietnam/NCVD-03/2008**	7.1	M	G	E	P	I	K	N	N	T	V	N	Q	K	S
**RG-A/chicken/Henan/12/2004**	9	Q	G	K	S	I	K	N	S	T	R	D	R	K	S

*Trivalent vaccine strains (A/Vietnam/1203/04, A/Indonesia/CDC669/06 & A/Anhui/1/05).

a Neutralizing epitopes characterized by Kaverin et al., 2007^16^.

b Neutralizing epitopes characterized by Prabakaran et al., 2010^2^.

In the present study, the HA genes of selected vaccine strains were engineered into a single MVA vector to generate the MVAtor-tri-HA vector. Further, immunogenicity and cross-protective efficacy of MVAtor-tri-HA vector was evaluated in a mouse protection model. The results showed that mice immunized i.m. twice with MVAtor-tri-HA vector induced robust serum HAI titer against homologous and heterologous clades (clade 4.0, 7.0 and 9.0) of H5N1 strains. In addition, MVAtor-tri-HA vector elicited potent cross-clade neutralizing antibody titers (>1∶735) against distinct clades of H5N1 strains compared to monovalent vaccines. In our previous study, three mixtures of HA expressed on the baculovirus with adjuvant showed comparatively lower neutralizing antibody titers (1∶220–240) of against similar clades of H5N1 strains [2]. The robust antibody response induced by the MVAtor-tri-HA vector compared to the baculovirus expression system could be due to the efficient in vivo delivery of HA genes by a single recombinant MVA vector. However, monovalent inactivated whole virus or MVAtor-mono-HA vector was able to induce a substantial neutralizing antibody response only against the homologous H5N1 strain.

The protective efficacy of the vaccine candidate was evaluated by challenging the immunized mice with a homologous (clade 1.0) and an antigenically distinct heterologous clade (clade 7.0) of H5N1 strains. The results demonstrated that mice immunized with MVAtor-tri-HA vector provided complete protection against 10 MLD_50_ of homologous or heterologous H5N1 challenge. However, our previous pilot study showed that mice immunized with MVAtor-mono-HA (A/Vietnam/1203/04) vector provided only moderate (66.6%) protection against 10 MLD_50_ homologous H5N1 strain (data not shown). In this study, mice immunized with adjuvanted inactive whole virus also showed rapid decline in body weight and showed insufficient protection against distinct H5N1 clades. This might be due to the variations in the neutralizing epitopes of hemagglutinin among the H5N1 strains. Also, we report that replication-deficient MVAtor vector expressing selected HAs induced robust neutralizing antibody response after two immunizations. These data indicate that pre-existing anti-vector immunity does not interfere with protective immune responses after immunization using the same MVA vector.

Also, the serological surveillance of post-immunized guinea pig sera against distinct H5N1 demonstrated that MVAtor-tri-HA induced robust cross-clade immunity against 20 heterologous clades or sub clades (clade 0, 1, 2.1, 2.2, 2.3, 4, 7, 7.1 and 9) of H5N1 strains, including the circulating strains from clade 1.1, 2.2.1.1 and 2.3.2.1. This observation shows the ability of the MVAtor-tri-HA vector against the genetic drift from 2010 to 2012 in clade 1.1 and 2 sublineages of currently circulating strains in Asia and Egypt (Table 1). Importantly, MVAtor-tri-HA vector induced high levels of neutralizing antibodies against H5N1 vaccine strains that have been recommended by WHO for vaccine development between 1997 to 2010 [20]. The results indicated that there was an excellent serological match between currently circulating H5N1 strains and the combination of selected vaccine strains. The neutralizing epitopes of identified HA1 region of the three HAs were also compared with different clades of H5N1 strains (Table 2). The results showed that neutralizing epitopes at amino acids 138, 140 and 141 in the 140 s loop, amino acids 154 and 155 in the 150 s loop and amino acids at position 162, 189 (H5 numbering excluding signal peptide) are located within the receptor binding site. The combination of three HAs shows the sufficient coverage of most variations in the neutralizing epitopes of HA1 region of different clades of H5N1 strains (Table 2). The cross-clade neutralizing efficiency of MVAtor-tri-HA could be due to efficient coverage of variations within the identified major neutralizing epitopes (Table 2) and also high affinity and avidity of the antibodies generated against conserved epitopes. Recently, Hu et al. [21] reported that an antibody against the conserved conformational epitope comprising amino acid residues at positions 118, 121, 161, 164, and 167 (H5 numbering) was cross-reactive with almost all H5N1 influenza strains.

In summary, the combination of three carefully selected HAs in a single vaccine candidate has efficiently induced neutralizing antibody titers against distinct clades of H5N1 strains isolated between 1997 and 2012. The use of a single multivalent MVA vector is the major advantage of this approach allowing cross-protection against a broad range of H5N1 strains with a single manufactured product. Thus, further investigations of the efficacy of multivalent MVAtor-HA vaccine candidates in ferrets and human are warranted.

In conclusion, trivalent MVA vector expressing three carefully selected HAs exhibited a robust cross-neutralizing antibody response that mediates protection against distinct H5N1 challenge. Further serological surveillance of post-vaccinated guinea pig sera demonstrated that MVAtor-tri-HA vector efficiently neutralized six distinct clades/subclades that emerged worldwide by natural evolution. Therefore it seems likely that the demonstrated broadly neutralizing efficacy of the MVAtor-tri-HA vector could also induce some degree of cross-protection against yet unknown pandemic H5N1 strains. The strategy for vaccine strain selection and vaccine candidate design indicates the feasibility of the development of a broadly protective ‘universal’ H5N1 vaccine for pandemic preparedness.
